# Quality of Life Measurement in Dogs and Cats: A Scoping Review of Generic Tools

**DOI:** 10.3390/ani12030400

**Published:** 2022-02-08

**Authors:** Annabelle E. Fulmer, Linda J. Laven, Kate E. Hill

**Affiliations:** 1 Maroubra Veterinary Hospital, 88 Bunnerong Rd, Pagewood, Sydney, NSW 2035, Australia; 2 Tāwharau Ora, School of Veterinary Science, Massey University, P.O. Box 11222, Palmerston North 4442, New Zealand; L.J.Laven@massey.ac.nz (L.J.L.); K.Hill@massey.ac.nz (K.E.H.)

**Keywords:** quality of life, quality of life measurement, questionnaire, dogs, cats

## Abstract

**Simple Summary:**

Quality of life (QoL) assessment tools play an important role in veterinary medicine by optimising companion animal welfare and directing treatment decisions. No distinct guidelines are currently available for tool design and appraisal. Nine published generic QoL assessment tools designed for use in dogs and cats were compared to each other. Each tool was uniquely individual in terms of structural design, psychometric evaluation and statistical analysis. Although each tool was unique, the majority assessed similar aspects of dog and cat QoL, namely activity level, the desire for interaction and appetite. These findings provide a reference point for future tool development by emphasizing the need for more consistency in reporting methodology and statistical validation, as well as highlighting potential valuable aspects of QoL in dogs and cats.

**Abstract:**

Quality of life (QoL) assessment in companion animals is an essential aspect of veterinary medicine that helps guide treatment decisions and ensures optimal animal welfare. Veterinarians and pet owners can use disease-specific or generic QoL assessment tools to evaluate an individual animal’s QoL. The aim of this scoping review was to identify and assess published generic QoL assessment tools suitable for use in either dogs or cats. A literature search identified 82 relevant publications, nine of which contained appropriate generic QoL assessment tools in accordance with inclusion and exclusion criteria. Each tool was assessed for evidence of psychometric evaluation including statistical analysis, reliability and validity. Commonly included items were determined to highlight potential important aspects of dog or cat QoL. Five of the nine publications used a statistical method such as factor analysis to determine tool design and structure. Although at least one aspect of reliability and validity was assessed for seven of the tools, none were validated across all measures. Two of the publications contained minimal to no statistical analysis. Common items for both dogs and cats included those regarding activity level, the desire for interaction and appetite. In addition, common items for cats included those regarding mood and grooming. This scoping review identified and evaluated currently available generic QoL assessment tools, providing a reference point for future tool development and validation.

## 1. Introduction

Due to the intense human–animal bond, pets are often considered valued and respected members of the family [1,2]. Throughout an animal’s life, a variety of health issues and medical conditions may develop requiring appropriate treatment or palliative care where necessary. Caring for a beloved pet with an illness or when approaching the end of their life can be challenging for many pet owners. When an animal is nearing death, either through old age or terminal illness, accurate and timely decisions surrounding hospice care and euthanasia are crucial to minimise discomfort and distress. To help guide the decision-making process and ensure optimal animal welfare, quality of life (QoL) must be taken into consideration [3]. Decisions involving appropriate treatment, when to treat, or when not to treat, all involve QoL assessments. Both veterinarian and owner can evaluate an animal’s QoL through subjective clinical assumptions in conjunction with QoL assessment tools.

QoL is a challenging concept to define due to its multi-faceted and subjective nature. Although most people have a general understanding of what QoL means, the term lacks a standardised method of measurement and consistent definition [4]. One of the reasons for this complexity is that many factors contribute to an individual’s QoL including health, psychological state, social relationships, environmental influences and external pressures [5]. The World Health Organisation (WHO) defines QoL as “an individual’s perception of their position in life in the context of the culture and value systems in which they live and in relation to their goals, expectations, standards and concerns” [6]. According to this definition, QoL is a personal, private and subjective experience of how an individual feels about his or her own life, rather than an external view of how others perceive it to be. It is determined not only by one’s life circumstances but also what uniquely matters to them and their ability to enjoy life [7].

As QoL is a concept which comes from within, assessments should be made from the perspective of the individual in question. However, this is not always achievable in human health care and is impossible within the veterinary profession [7]. Often in the form of a questionnaire or scale, QoL assessment tools in humans are designed to be self-reported where possible (patient-reported outcome measures (PROs)). In those unable to self-report, such as young children or the cognitively impaired, an observer or proxy (i.e., parent/clinician) is required to assess QoL on the individual’s behalf (observer-reported outcome measures (OROs)) [8,9]. In the veterinary profession, as animals cannot directly express how they feel, all QoL assessment tools are classified as OROs [7]. Therefore, it is the responsibility of the veterinarian and owner to estimate a pet’s QoL based on criteria deemed meaningful to that individual animal [10].

In the human health care system, QoL assessment tools have been extensively developed and widely used for decades [8,9]. Yet in comparison, research and development of these tools in the veterinary profession is relatively within the early stages [11,12,13]. In 2009, in response to the demand for accurate PROs to support product labelling claims within human medicine, the US Food and Drug Administration (FDA) issued a ‘Guidance for Industry’ document outlining the psychometric properties required to produce a valid and reliable QoL assessment tool [14]. Validity refers to whether a tool measures what it claims to measure, whereas reliability is the repeatability of a tool [15]. As outlined in the FDA guidelines, numerous types of validation are available depending on tool design and structure. Demonstrating good validity and reliability is paramount before using a tool in a clinical context to ensure accurate results. While the FDA guidelines were intended for validating human PROs, they are based on well-established psychometric principles also relevant to OROs and as such can be applied to veterinary QoL assessment tools [16].

The benefit of QoL assessment in human health care is clearly evident. Within research QoL assessments are useful in evaluating health care interventions and treatments, identifying health inequalities, understanding the burden of disease and assisting in allocating resources [9]. In clinical practice, these tools help identify and prioritise problems, improve communication between patients and health care workers, assist in clinical decision making and monitor symptoms/side effects in response to treatment [17]. Furthermore, QoL assessments minimise the risk of incorrect assumptions surrounding an individual’s QoL following a procedure or treatment protocol. For example, Sugarbaker et al. [18] assessed QoL in humans undergoing treatment for soft tissue sarcoma of the limb. They assumed patients would prefer to keep both legs, and therefore hypothesised a higher QoL in those receiving limb-sparing surgery/radiation compared to amputation. However, by using validated QoL assessment tools, they found patients who underwent limb amputation actually reported a much higher QoL in regard to function, mobility, psychosocial and economic impact, as well as sexual function and pain. This finding demonstrates the importance of QoL assessment not only in humans but also in veterinary patients, as similar incorrect assumptions may occur regarding an animal’s QoL.

QoL assessments provide a quantitative guideline for veterinarians and owners to openly and honestly evaluate a pet’s QoL to assist in both treatment and euthanasia decisions [13]. They help owners understand the severity of their pet’s health issues and the impact on his or her ability to enjoy life. By regularly referring to these tools, systematic assessments regarding an animal’s QoL can be achieved and a potential decline in well-being may be identified early on [5]. Research suggests that these assessments encourage owners to think about their pet’s well-being and provide support for decisions regarding veterinary care. Mullan et al. [19] developed a QoL screening tool in the form a questionnaire to raise awareness of welfare in pet dogs. After completing the questionnaire, several participating owners found the tool encouraged them to think about and subsequently improve their dog’s QoL. Similarly, Mwacalimba et al. [2] found a canine QoL survey to be of value in a primary veterinary clinic, with 81% of pet owners expressing interest in learning about QoL.

In both human and veterinary medicine, two categories of QoL assessment tools are recognised. Firstly, there are disease-specific tools that relate to individual health issues, and secondly there are generic or holistic tools that globally assess QoL. The majority of QoL assessment tools in veterinary medicine are disease specific [20]. Such tools have been developed for use in dogs with atopic dermatitis [21], spinal cord injury [22], cancer [23], and obesity [24] and in cats with diabetes [25], chronic kidney disease [26] and heart disease [27] to name a few. The main advantage of disease-specific tools is that they allow for a detailed and precise quantification of how a pet’s QoL is impacted by a particular disease and/or treatment. The main disadvantage is that they are not suitable for all animals and do not allow for comparisons across species and diseases [12]. Generic or global QoL assessment tools overcome that disadvantage as they can be used with any animal or disease and also allow for comparisons. As they do not provide a detailed QoL assessment regarding a specific disease or treatment, generic tools are more robust for those animals with multiple co-morbidities, such as geriatric pets nearing the end of their life.

Generic QoL assessment tools within the veterinary profession are an important means to assist in both treatment and end of life decisions. The aim of this scoping review was to identify and evaluate all currently available published generic QoL assessment tools designed for use in either dogs or cats.

## 2. Materials and Methods

### 2.1. Definition of Terms

A quality of life (QoL) assessment tool was defined as a set of questions designed for pet owners and veterinarians to measure the QoL of either cats or dogs. The tool could be in the form of a scale or questionnaire and contain an indefinite number of domains or items. A domain was defined as a broader term to be measured such as appetite, whereas an item referred to a specific question within that domain such as ‘How much does your cat enjoy food?’. A QoL assessment tool was considered general if it was designed to assess QoL as a whole, rather than focusing on one particular condition.

### 2.2. Search Methods

This scoping literature review followed the Preferred Reporting Items for Systematic reviews and Meta-Analyses (PRISMA) flowchart and is in accordance with PRISMA’s statement [28]. A keyword search of the literature was performed to determine all available existing publications related to QoL assessment tools and euthanasia decision making in companion animals. Scopus (1980–2020), PubMed (1987–2020; MEDLINE) and all electronic databases within Web of Science (1980–2020; Web of Science Core Collection, BIOSIS Previews, Current Contents Connect, Chinese Science Citation Database, CABI: CAB Abstracts, KCI- Korean Journal Database, MEDLINE, Russian Science Citation Index, SciELO Citation Index, Zoological Record) were searched during May 2020 to identify all relevant peer-reviewed literature. Default date ranges for each database were used during the search process. Searches using the following terms were performed with keywords linked via Boolean terms:

((“quality of life”) AND (decision* OR assess*) AND (dogs OR canine OR cats OR feline) AND (questionnaire* OR tool OR scale OR measure* OR survey) AND (veterinar* OR “small animal practice”))

### 2.3. Inclusion Criteria

The inclusion criteria for each publication were as follows: (1) be written in English; (2) contain at least one keyword within the abstract of the article; (3) contain a tool designed to assess the general QoL of either dogs or cats; (4) be the original publication of that tool; and (5) be readily available or adequately described either within the body of the publication or accessible free of charge.

### 2.4. Exclusion Criteria

The exclusion criteria for each publication were as follows: (1) not written in English; (2) did not contain one of the keywords within the abstract of the article; (3) did not contain a tool to assess the general QoL of either dogs or cats; (4) contained only a disease-specific QoL assessment tool; (5) the tool was not described within the publication or was only available for a monetary fee.

### 2.5. Selection Process

**Figure 1:**
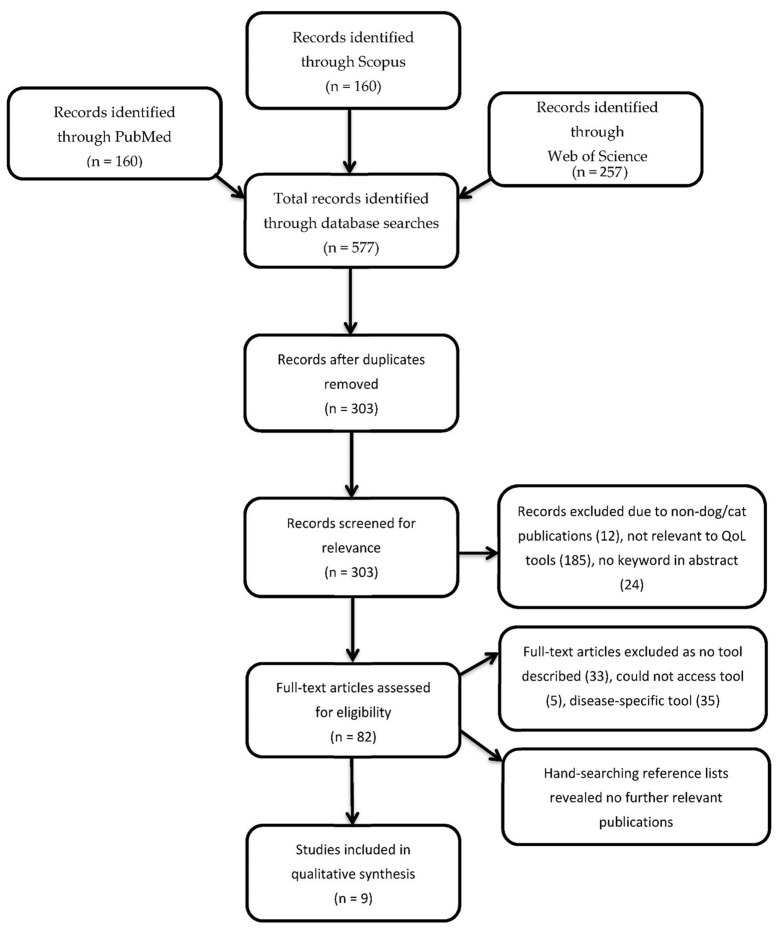
Summary of the systematic approach to the literature using a PRISMA flow diagram [28].

The initial search process, the application of inclusion and exclusion criteria and the assessment of eligible publications were performed independently by a single author (AF). Following a keyword search of the literature, the titles and abstracts of identified publications were screened for relevance to remove those unrelated to QoL assessment tools. To determine eligibility, inclusion and exclusion criteria were applied to all relevant publications. Where applicable, decisions involving whether to include debatable publications were made independently by the second (LL) and/or third (KH) authors. Publications were organised using the reference manager Endnote X9 (Clarivate Analytics) [29]. For those publications which met relevant inclusion criteria but the instrument was not adequately described or accessible online, authors were contacted via email. If authors failed to respond within six weeks, the publication was excluded. Follow up emails were not performed. Hand searching reference lists of eligible publications was performed to screen for relevant studies which may have been missed through the initial search process. A PRISMA flow diagram was completed to record the numbers of all the articles in this scoping review (Figure 1) [28].

### 2.6. Evaluation of Psychometric Properties; Statistical Analysis, Reliability and Validity

Each QoL assessment tool was evaluated independently by a single author (AF) for evidence of statistical analysis, reliability and validity (Table 1). Using criteria outlined in the US FDA guidelines for human PROs [14], the presence or absence of validation was determined for each tool. Specifically, reliability was demonstrated by internal consistency and/or test–retest reliability, whereas validity was demonstrated by content, criterion and/or construct validity. Evidence for statistical analysis was determined in terms of the reported factor analysis (FA) or principal component analysis (PCA).

### 2.7. Evaluation of Items

Items within each QoL assessment tool were evaluated independently by a single author (AF) to determine those most commonly included. Comparisons among items were made between all selected publications and between species-specific tools. An item was considered common if it was included in ≥75% of the QoL assessment tools.

## 3. Results

The search returned 577 publications, of which 82 were found appropriate after screening titles and abstracts for relevance (Figure 1). Following the application of inclusion and exclusion criteria to 82 relevant articles, nine publications remained. These publications dated from 2005 to 2019 and appeared in six distinct journals.

Each of the nine publications included one generic QoL tool, the details of which are described in Table 2. Four of these tools were designed for use in cats, four for use in dogs and one for use in both dogs and cats. Two tools were readily accessible online [16,26], two were available from the author on request [19,30] and the remaining five were adequately described within the publication. The tools ranged from a simple one-page scale containing seven items [31] to a multi-page questionnaire containing 39 items [19]. Six of the nine tools contained items answered solely via Likert scales, whereas the other three contained items answered either via visual analogue scales (VAS) [31], interval scales [30] or a mixture of both VAS and Likert scales [19].

### 3.1. Evaluation of Psychometric Properties; Statistical Analysis, Reliability and Validity

The types of validity, reliability and whether statistical analysis was performed for each tool are summarised in Table 1. Of the nine publications, seven described a study in which a draft QoL tool was completed by a sample of pet owners and subsequently assessed for reliability and/or validity. Following assessment, six of these tools underwent adjustments to remove items and rearrange domains. One publication [31] used two randomised controlled trials to evaluate a QoL assessment tool, whereas another [13] simply described a tool without performing a study or further analysis. Five of the nine publications used a statistical method such as factor analysis to determine the design and structure of the initial QoL tool. Although at least one aspect of reliability and validity was assessed for seven of the tools, none were validated across all measures as criterion validity and inter-rater reliability were not evaluated.

### 3.2. Evaluation of Items

Each of the nine final QoL assessment tools were compared to establish the most commonly included items (Supplementary Tables). Regardless of whether designed for use in dogs or cats, all nine QoL tools included an item responsible for assessing a pet’s activity as well as their desire for interaction with either humans or their surroundings. Seven of the nine tools included an item responsible for assessing appetite. In addition, for those tools designed for cats, all four contained an item responsible for assessing a cat’s mood, whereas three tools contained an item responsible for assessing grooming. Four of the nine QoL tools included a broad question designed to assess the general QoL of the pet.

## 4. Discussion

This scoping review used a systematic approach to assess published generic QoL assessment tools currently available for use in small animal veterinary medicine. Although various tools were identified within the literature, only nine generic tools intended to assess global QoL met inclusion and exclusion criteria. Four tools were designed specifically for use in dogs, four for cats and one for both species. Compared to human health care, development and research of these tools within the veterinary profession are still within the primary stages (11). No distinct guidelines are currently available for tool design and appraisal. Consequently, each tool was uniquely individual in terms of structural design, psychometric evaluation and statistical analysis.

The primary goal of a QoL assessment tool is to gain an understanding of an animal’s well-being to help guide decisions involving health care and appropriate treatment [7]. Not only are these tools useful for QoL assessment of terminally ill patients in palliative care, they also screen for medical conditions, identify health issues early on, monitor changes over time and measure response to medical intervention [5]. Due to these varied uses, the intended purpose and design of each of the nine generic tools evaluated in this review was quite diverse. For example, the tool described by Villalobos [13] was developed primarily to aid decisions surrounding palliative or hospice care, whereas Yeates et al. [31] designed a tool to be used during veterinary consultations to promote discussions regarding quality of life. Some tools were designed to be taken online within 10–15 minutes [32], others were completed within a 15–30 minute veterinary consultation [19] and one tool required a 60 minute telephone interview [30]. The diversity among the nine tools is a reflection of the varied uses for QoL assessment within veterinary medicine and reinforces the value for such tools within the profession.

Comparisons between the nine tools determined the most commonly included items. If an item was referenced in the majority of tools following psychometric evaluation, it was considered popular with good psychometric properties and therefore a potentially valuable representation of a pet’s QoL. Although more recently published articles such as Noble et al. [33] may have referred to previously published QoL tools, item generation for each publication was commonly determined via the use of discrete focus groups consisting of dog/cat owners, veterinarians and animal welfare specialists. The use of distinct groups of people to generate items for each individual tool largely accounted for the potential repetition of items from previously published tools. As each tool was uniquely different and consisted of a variable number of domains and items, direct comparisons were challenging and sometimes impractical. Due to the diversity among the grouping of items, the nomenclature of domains was vastly dissimilar for each tool. As a result, similarities among items were determined, as opposed to similarities among domains.

All nine QoL assessment tools included items related to the activity of an animal. For example, Lavan [32] included items such as “My pet is as active as he/she has been” and “My pet moves normally”. Similarly, all nine tools included at least one item regarding an animal’s desire for interaction with humans or their surroundings. For example, Freeman et al. [16] included items asking “My cat greeted me when I returned from being away” and “My cat was curious about his/her surroundings”. Items relating to appetite were also common among seven of the QoL assessment tools. For example, Wojciechowska et al. [30] included an item asking dog owners “Thinking about the last seven days, would you say that _____ enjoyed his food?”. Further similarities among items were determined for those four QoL assessment tools designed only for cats. All four contained an item relating to mood and three contained an item regarding grooming behaviour. For example, Tatlock et al. [34] included an item asking “In the past 4 weeks my cat has appeared happy” and Bijsmans et al. [26] asked “How has your cat’s grooming been during the past week?”.

These findings suggest that three aspects of QoL, namely activity level, the desire to interact and appetite, appear to be valuable QoL parameters for both dogs and cats. In addition, mood and grooming behaviour also appear to be important aspects of a cat’s QoL. Other studies evaluating QoL parameters using disease-specific tools are supportive of these results. Research by Tzannes et al. [35] and Reynolds et al. [36] found that a cat’s appetite and desire for interaction were among the most mentioned parameters when owners of cats with lymphoma [35] and heart disease [36] were asked about QoL influences. Similarly, Oyama et al. [37] found owners of dogs with heart disease considered their dog’s desire to interact with them as the most important indicator of QoL. By highlighting valuable QoL parameters within the present review, future studies designing global QoL assessment tools for dogs and/or cats may consider including equivalent domains and items.

However, due to the small sample size of only nine QoL assessment tools, interpreting these results requires caution. Although items relating to activity and the desire to interact may be valuable QoL parameters, other aspects not covered adequately in these particular tools may be just as important. For example, only five of the nine tools included at least one item relevant to pain. Of the 27 items included in the tool published by Wojciechowska et al. [30], only one assessed pain. The authors acknowledge that by only including one item relevant to pain, misguided conclusions may occur regarding the significance of that aspect to QoL. From a clinical perspective, pain is likely a very important consideration of QoL for any species. When not effectively treated in humans, pain can lead to a damaging effect on all aspects of QoL [38]. A study by the WHO showed that people with persistent pain are much more likely to have depression, anxiety and difficulty working compared to those without pain [39]. Pain is a challenging construct to evaluate in animals due to its multi-faceted and subjective nature. Measuring acute and chronic pain in pets is often based on behavioural observation by the veterinarian and/or owner [40]. In regard to acute pain, scales measuring facial expressions such as the Feline Grimace Scale (FGS) have demonstrated good reliability and validity [41]. Future research regarding QoL assessment tools may consider including pain, perhaps in the form of a grimace scale, to determine its significance and psychometric properties.

Four of the nine QoL assessment tools included a single question regarding the overall QoL of the animal. For example, Lavan [32] included a single item asking owners to rate their dog’s overall QoL answered via a 10-point Likert scale (1 = very poor; 10 = excellent). Inclusion of a single global question regarding QoL is common practice in human health care [42]. It not only provides additional insights into an individual’s perception of their own QoL but also facilitates brief, meaningful dialogues between patient and clinician [42,43]. By asking a single global QoL item, some authors have proposed that useful responses may be generated by encouraging pet owners to pause and reflect on their own animal’s QoL [44]. In the present study, both Freeman et al. [16] and Tatlock et al. [34] found a single global QoL item to be of benefit in supporting the validity of their tools. Both these publications found strong positive correlations between the global QoL score and the overall score of the tool, i.e., cats scoring higher on the global QoL item by their owner had higher overall scores. However, not all publications that included a global QoL question found it particularly useful. Bijsmans et al. [26] asked the question “On a scale from one to ten, 1 being a very poor quality of life and 10 being an excellent quality of life, I feel that my cat’s quality of life during the past week was…”, which they correlated with the tools final score. Only a moderate correlation was found (*p* = 0.52) between the global question and the final score, suggesting that the global question incompletely assessed a cat’s QoL. Even though Bijsmans et al. [26] found only a moderate correlation, it may still be worthwhile including a global QoL question to encourage pet owners to consider their animal’s QoL from a different perspective. For example, Mullan et al. [19] included a single question asking owners ‘how willing would you be to take on the life your pet is now living?’. By answering such a simple, direct question, many owners reported that it gave them a new way to think about their dog’s life.

Another consideration is whether it may be beneficial to include an item or domain that takes into account the QoL of the pet owner or primary caregiver [12]. For those geriatric or ill pets, owners may need to be heavily involved in husbandry or care which may negatively impact the owner’s QoL. A study by Niessen et al. [25] demonstrated that owners of diabetic cats found twice daily insulin injections significantly decreased the owner’s QoL, even more so than the perceived QoL of the cat. In human medicine, OROs designed for babies and infants often incorporate items and domains regarding the parent’s QoL in terms of the impact on time limitations, family coherence and mental health [45]. Including items and domains assessing the pet owner’s QoL may highlight potential negative effects, resulting in the consideration of different treatment options or even euthanasia.

The psychometric approach to designing a QoL assessment tool firstly involves generating numerous items and domains through interviews with pet owners and experts in the field such as veterinarians or animal welfare advocates. Following pilot testing, the design and structure of the tool are established by reducing these items and grouping them together using statistical techniques such as factor analysis or principal component analysis [15]. Further statistical validation is then required to assess validity and reliability. In accordance with the US FDA guidelines for PROs [14], psychometric properties including statistical analysis, validity and reliability were determined for each of the nine published tools.

Although not all publications followed a psychometric approach, the generation of items and domains for each of the nine tools all involved the use of pet owners and/or veterinarians. This initial step in tool design is vital to ensure a high standard of quality and establish content validity [42,44]. In regard to a QoL assessment tool, content validity refers to whether items and domains adequately cover and comprehensively measure QoL. In the development of PROs, patients must be involved in item generation to establish content validity [14]. However, as all veterinary QoL tools are OROs, proxies such as pet owners or veterinarians are required.

Following item generation, five of the nine QoL assessment tools underwent psychometric evaluation using factor analysis or principal component analysis to determine tool structure and design. Statistical techniques such as factor analysis are essential to identify items that correlate well together and eliminate problem items which lack clarity or fail to discriminate between respondents [15]. Reducing and refining items using factor analysis creates a more structured and concise QoL assessment tool containing good psychometric properties. However, clinical sensibility and subjective judgment are still required to retain items which are thought to measure QoL despite poor psychometric analysis [46]. In comparison to the four tools which lacked such statistical methodology, the five tools subjected to factor analysis or principal component analysis were more recently published between 2013 [32] and 2019 [33]. The current demand for more statistically validated QoL assessment tools within the veterinary profession may be a reason why the psychometric approach is mostly evident in more recently published tools [20].

To have confidence in a QoL assessment tool, it must consistently measure what it claims to measure and produce accurate results over multiple trials. In short, the tool must be both valid and reliable [47]. Seven of the nine QoL assessment tools underwent statistical validation and demonstrated a combination of at least one type of reliability and one type of validity. In terms of reliability, measures included in the publications consisted of internal consistency and test–retest reliability. Internal consistency determines whether correlations exist between items grouped together within a domain, whereas test–retest reliability measures the tools ability to produce consistent results when administered to the same person on two separate occasions [14]. Internal consistency was adequate across the five publications that included the measure, whereas test–retest reliability was adequate in five of the six publications that included the measure. Wojciechowska et al. [30] found their tool had poor test–retest reliability that could have resulted from a number of factors including poorly worded items, a change in the dog’s health during the test–retest period or a ‘response shift’ in which an owner alters their response despite no change in their animal’s QoL [48].

In terms of validity, measures included content validity and construct validity. As mentioned previously, all tools demonstrated clear evidence of content validity. However, only seven of the tools also assessed construct validity. Construct validity refers to the extent to which the tool conforms to existing hypotheses or theories concerning relationships among items or domains [47]. For example, one could hypothesise that sick cats may have a poorer QoL compared to healthy cats; therefore, a tool with good construct validity would score sick cats with poor QoL as having lower QoL. Due to differences in study design, the hypotheses for measuring construct validity were unique for each tool making direct comparisons complicated. Nevertheless, the seven publications that assessed construct validity reported the measure as acceptable.

Additional measures of reliability and validity not assessed in any of the nine publications include criterion validity and inter-rater reliability. For a tool to have good criterion validity, responses must correlate with those of a ‘gold standard’ [49]. A likely reason criterion validity was not measured in any publication is that a gold standard for assessing global QoL in animals currently does not exist [33]. To demonstrate good inter-rater reliability, the tool must produce similar results when administered by multiple examiners [50]. Consequently, more than one pet owner is required to know a particular animal’s QoL. A potential reason for not assessing inter-rater reliability in these publications is that the majority of animals included in the samples may have belonged to single-person households. To measure inter-rater reliability, those animals living in multi-person households would require identification and individual assessment. An important consideration when assessing shared households is the potential difference in gender perceptions of an animal’s QoL. When male and female owners were presented with a questionnaire regarding their own dog’s perceived levels of stress, men were more likely to consider their dog as showing low levels of stress, whereas women were more likely to consider their dog as being moderately stressed [51]. Future research assessing the inter-rater reliability of a QoL tool in multi-person households should consider potential gender differences in QoL assessment.

Two tools described in the current review, namely Yeates et al. [31] and Villalobos [13], contained minimal evidence of statistical validation. Content validity was evident as veterinarians contributed to the generation of items and domains but all other measures were absent. The lack of statistical validation likely reflects the objective and design of each publication. The aim of the tool described by Yeates et al. [31] was to promote QoL discussions within a veterinary consultation between client and veterinarian. As such, the design of the study was to assess whether the tool promoted QoL discussions, as opposed to whether it measured QoL itself. The tool described by Villalobos [13] was created by the author to assist pet owners and veterinarians in end of life decisions. The objective of the publication was to simply describe a QoL assessment tool, rather than demonstrate validity and reliability. As such, the structure of the tool consisted of broad open domains for contemplation, as opposed to specific items, making it unsuitable for statistical validation. However, to be certain these two tools accurately measure QoL, statistical validation would be required before use in a clinical setting.

For a QoL assessment tool to be clinically useful, it must be able to accurately establish declining QoL and differentiate between animals with opposing QoL. Two of the nine QoL assessment tools determined whether the tool could differentiate between groups of animals in which the authors expected to differ. For example, Bijsmans et al. [26] compared three groups of cats to one another, young healthy (YH), old healthy (OH) and those with chronic kidney disease (CKD). As hypothesised by the authors, cats in the YH group scored higher on the QoL assessment tool compared to cats in the OH or CKD group, suggesting that their tool could differentiate between healthy and sick cats. Similarly, Noble et al. [33] suggested that their tool could also differentiate between healthy and sick cats as overall QoL scores were significantly higher in healthy cats for all three domains. However, QoL is not the same thing as health. Although health status may affect QoL, animals of an older age or those diagnosed with an illness such as CKD may in fact have higher QoL compared to young healthy animals. Unless the QoL of each individual healthy and sick animal is known, comparisons between such groups using a generic QoL tool may not be accurate. Furthermore, during the initial stage of tool development, Noble et al. [33] found that nearly 30% of pet owners reported their cat as healthy when veterinarians deemed them sick. This finding is very significant as Bijsmans et al. [26], along with other tools included in this review [16,32,34], recruited healthy animals determined solely by their owners. Without physical examinations or diagnostic testing to verify the animal’s health, it is uncertain whether these supposed healthy groups of animals were in fact healthy. Future research into whether a QoL assessment tool accurately differentiates between healthy and sick animals with opposing QoL should consider veterinary consultations and examinations to verify an animal’s health.

There are several limitations to the current review. As mentioned earlier, a small sample size of nine tools requires caution when interpreting results, especially in terms of commonly included items. Additional tools would be required to increase confidence in results. Although some eligible publications appeared promising, they contained incomplete or partially reported tools. Emails were sent to authors requesting further information; however, as a few did not respond, they were not included in the present study. Follow up emails may have been beneficial to remind the authors to respond. Lastly, the initial search included the use of three databases, Web of Science, PubMed and Scopus. Additional literature may have been available elsewhere and therefore missed in the search process.

Another limitation of the present study was that a single author was responsible for reviewing the literature. To reduce the possibility of discarding relevant publications, at least two reviewers are recommended to independently screen the literature with any discrepancies resolved through consensus or a third party [52]. Although two or more reviewers is the gold standard, Nama et al. [53] suggest that by applying multiple relevant exclusion criteria, publications could be excluded by a single reviewer without loss of sensitivity. As both multiple inclusion and exclusion criteria were applied to the screening process in the current study, sensitivity may have been retained.

A major challenge and limitation with veterinary QoL assessments is that they are all OROs requiring proxy assessments [7]. As animals cannot themselves contribute to QoL tools, people who are most familiar with the animal in question, such as pet owners and caregivers, are responsible for making QoL evaluations. By using an ORO, we are assuming the pet owner is capable of observing, comprehending and interpreting their animal’s behaviour, an assumption that may not always be true. In human medicine, PROs are preferred to OROs, as proxy assessments are not always accurate [8,14,54]. McCusker et al. [54] compared responses to a general QoL measure of close relatives who were acting as proxies with those of terminally ill people. They found that proxies consistently underestimated the ill patient’s QoL, considering them more impaired than what the patients considered themselves to be. As such, OROs may never accurately reflect an individual animal’s life experience [12]. Nevertheless, these limitations should not discourage veterinary medicine from designing and evaluating QoL assessment tools.

## 5. Conclusions

QoL assessment is an important aspect of veterinary medicine [11]. At present, there are no veterinary guidelines available for research and development of these tools. Consequently, limited numbers of published generic QoL assessment tools suitable for use within a clinical setting exist. Nine generic tools reviewed in the current study demonstrated similarities among items that may be of benefit for future tool development. However, the process of psychometric evaluation was very different for each tool, highlighting the need for more consistency in reporting methodology and statistical validation. QoL assessment is a rapidly evolving field that requires additional tool development to assess generic QoL. To be of greater use within clinical practice, there is a need for further research to validate newly developed tools and refine those already in existence.

## Figures and Tables

**Table 1:** Assessment of psychometric properties including statistical analysis, reliability and validity of the nine published QoL tools (adapted from the United States Food and Drug Administration [14]).

Measure	Test	Definition of Test	Noble et al. (2019)	Tatlock et al. (2017)	Freeman et al. (2016)	Bijsmans et al. (2016)	Lavan (2013)	Yeates et al. (2011)	Villalobos (2011)	Mullan et al. (2007)	Wojciechowska et al. (2005)
Statistical analysis to determine tool structure	Factor analysis (FA) or principal component analysis (PCA)	FA identifies correlations between and among variables (i.e., items) to organise them into underlying factors (i.e., domains). PCA estimates proportions of variance within a set of variables (i.e., items) to transform large amounts of data into smaller sets.	✓	✓	✓	✓	✓				
Reliability	Internal consistency	Determines how well items within the tool measure the same construct. Assessed using statistical measures (e.g., Cronbach’s alpha) to determine whether correlations exist between items grouped together within a domain.		✓	✓	✓	✓				✓
	Test–retest reliability	Indicates whether the tool yields consistent results when administered to the same person on two separate occasions.	✓	✓	✓		✓			✓	✓
	Inter-rater reliability	Agreement among responses when the tool is administered by two or more scorers.									
Validity	Content validity	The extent to which the tool measures the construct it is designed to measure. Can be established if the target population (i.e., pet owners) or panel of experts (i.e., veterinarians) contribute to item generation and cognitive interviewing.	✓	✓	✓	✓	✓	✓	✓	✓	✓
	Criterion validity	Determines whether responses to items agree with a ‘gold standard’ (i.e., an external criterion known to measure the same concept).									
	Construct validity	The extent to which items or domains measure what they are meant to be measuring. Assessed by determining whether relationships among items or domains conform to existing hypotheses or theories (i.e., known-group validity, convergent validity).	✓	✓	✓	✓	✓			✓	

**Table 2:** Summary of information extracted from the nine published QoL tools (continued on following page).

Publication	Species	Function of Tool	Development of Tool	Testing of Draft Tool	Description of Final Tool
Noble et al. 2019	Cats	Generic QoL tool for cats	Semi-structured interviews with owners of healthy and sick cats to generate 165 items. An online survey was completed by cat owners and vets to screen for relevance and clarity. Draft questionnaire consisted of 39 items.	Field test one: 71 cat owners (*n* = 41 sick, *n* = 30 healthy) completed the draft questionnaire online. Field test two: 94 cat owners (*n* = 58 sick, *n* = 36 healthy) completed the revised questionnaire online. In total, 48 of these owners completed the questionnaire a second time two weeks later.	Three domains (vitality, comfort and emotional well-being) with 20 items. Items answered using 7-point Likert scales. A final question ‘Is your cat perfectly healthy?’ was included, but was not assessed in the study. An algorithm was used to generate three domain scores to determine a cat’s QoL (algorithm not described by authors).
Tatlock et al. 2017	Cats	Tool for evaluating QoL in cats for use in general veterinary practice	Cat owners (*n* = 45) conducted an online survey followed by cognitive debriefing interviews (*n* = 10) to generate a draft QoL containing 22 items.	A sample of 199 owners of healthy cats completed the draft questionnaire online twice, two weeks apart.	Two domains (healthy behaviours and clinical signs) with 16 items. Items answered using 5-point Likert scales. An algorithm was used to generate QoL scores; 0 (lowest QoL) to 100 (highest QoL).
Freeman et al. 2016	Cats	Generic QoL tool for cats	Focus groups of cat owners (*n* = 23) developed 155 items, followed by cognitive debriefing interviews with novel cat owners (*n* = 31). Draft tool (Cat Health and Well-Being (CHEW) questionnaire) consisted of 100 items.	Sample of 1303 owners of healthy cats completed draft CHEW online; 391 of these owners completed it a second time 7 days later.	CHEW. Eight domains (mobility, emotion, energy, engagement, eyes, coat, appetite and fitness) with 33 items in total. Items answered using 6-point Likert scales plus N/A option. Contained two broad questions regarding 1) owner’s perception of a cat’s overall QoL and 2) a cat’s overall health. Computer-generated QoL score ranged between 0 (lowest QoL) to 100 (highest QoL).
Bijsmans et al. 2016	Cats	Generic QoL tool for cats. Also used to compare healthy cats to those with chronic kidney disease (CKD)	Focus group of vets (*n* = 5), vet nurses (*n* = 2) and cat owners (*n* = 32) developed 18 items included in draft tool (CatQoL).	Sample of 204 cat owners completed CatQoL online. Compared three groups of cats; old healthy (*n* = 35), young healthy (*n* = 94) and chronic kidney disease (CKD) (*n* = 70).	CatQoL. Four domains (general health, eating, behaviour and management) with 16 items. Items answered using 4-point or 7-point Likert scales. Contained one broad question regarding general QoL (1 = very poor; 10 = excellent). Computer-generated average-weighted impact score (AWIS) provided QoL measure.
Lavan 2013	Dogs	Generic QoL tool for dogs, developed for use in clinical and research settings	Owners of healthy dogs (*n* = 60) reviewed a cancer treatment survey developed by the author to determine appropriate items. Draft canine health-related QoL survey (CHQLS-21) consisted of 21 items.	Sample of 174 owners of healthy dogs completed the draft CHQLS-21 online; 86 of these owners completed it a second time 2 weeks later.	CHQLS-15. Four domains (happiness, physical functioning, hygiene and mental status) with 15 items. Items answered using 5-point Likert scales. Contained two open ended questions on general health and an item asking for a direct QoL assessment (1 = very poor; 10 = excellent). Computer-generated QoL score for each domain was determined by the sum of item scores.
Yeates et al. 2011	Dogs	Tool was designed to encourage discussions and decisions about dogs’ QoL	An expert panel of dog owners, vets and welfare scientists (*n* = 7) generated the QoL tool.	Two randomised controlled trials in which 170 owners were either given the tool during a consult (tool group, *n* = 91) or not (control group, *n* = 79). Dog owners were questioned after the consult regarding QoL discussions and decisions.	Seven items (food, exercise, comfort, company of humans, company of other dogs, routine veterinary care and care of illnesses) answered via visual analogue scales. No QoL score generated.
Villalobos 2011	Dogs and cats	Provides a guideline for caregivers of terminally ill pets to assess QoL and assist in end of life decisions	Author developed HHHHHMM QoL scale.	None	Seven domains (hurt, hunger, hydration, hygiene, happiness, mobility and more good days than bad). Variable number of items per domain Each domain answered using a 10-point Likert scale. A score of >35 deemed acceptable QoL.
Mullan and Main 2007	Dogs	Generic QoL tool to raise awareness regarding the welfare of pet dogs visiting a veterinary clinic	Expert focus group of vets, animal welfare specialists and behaviourists (*n* = 15) revised/refined a QoL questionnaire developed by the author.	Sample of 27 dog owners completed the questionnaire twice on two consecutive days.	Four part questionnaire containing 39 items. Part 1: personality and history. Part 2: 16-point Likert scale questions regarding comfort, exercise, diet, mental stimulation and companionship. Part 3 and 4: 22 visual analogue scale questions regarding behaviour. A concluding question required owners to list three important changes they would make to improve their dog’s QoL. No single QoL score generated.
Wojciechowska et al. 2005	Dogs	Discriminative questionnaire for assessment of non-physical aspects of QoL of pet dogs	Items were generated by the authors using personal veterinary experience and literature regarding animal welfare, veterinary medicine and canine behaviour. A focus group of five dog owners were also consulted. A panel of nine experts reviewed the draft questionnaire which consisted of 38 items.	A total of 120 dog owners (*n* = 43 healthy dogs, *n* = 77 sick dogs) completed the questionnaire via telephone interview. Owners of healthy dogs (*n* = 43) were reinterviewed three to four weeks later.	Five domains (satisfaction of telos needs, opportunities for pleasure, minimal fear and distress, distressing events and being outdoors) consisting of 27 items. Response options were assigned the letter grades O, A, B and C, (O = best QoL; C = worst QoL). These grades were designated the values 4 (O), 3 (A), 2 (B), and 1 (C) on an interval scale. QOL score = (mean grade/4) X 100%. The maximum possible score was 100%, whereas the minimum possible score was 25%.

## Supplementary Materials

The following are available online at https://www.mdpi.com/article/10.3390/ani12030400/s1, Table S1: Examples of commonly included items for each of the nine QoL assessment tools. Table S2: Examples of commonly included items for the four QoL assessment tools designed for cats.
