# Supplementary material for: Quality of Life Measurement in Dogs and Cats: A Scoping Review of Generic Tools
Source: Animals (Basel). 2022 Feb 8;12(3):400. doi: 10.3390/ani12030400 (PMC8833627; doi:10.3390/ani12030400)
Supplement: Supplementary file 1 [file animals-12-00400-s001.zip › animals-1524087 - supplementary.pdf]

Supplementary data

Table S1. Examples of commonly included items for each of the nine QoL assessment tools.

Potentially valuable aspect of QoL	Noble et al (2019) [30]	Tatlock et al (2017) [31]	Freeman et al (2016) [16]	Bijsmans et al (2016) [26]	Lavan (2013) [32]	Yeates et al (2011) [33]	Villalobos (2011) [13]	Mullan and Main (2007) [19]	Wojciechowska et al (2005) [34]
**Activity**	Please tell us how well this word describes your cat as he is today: Active.<br><br>7-point Likert scale (0 = Couldn't be more active, 6 = not at all active)	In the past 4 weeks my cat has been inactive or has had no energy	Thinking about just the last seven days….. my cat was full of energy/lively/seemed tired<br><br>6-point Likert scale (1 = always, 6 = never)	General health. Q4a. How active was your cat during the past week?<br><br>4-point Likert scale (1 = very active, 4 = very inactive)	Physical functioning. My pet is as active as he/she has been<br><br>5-point Likert scale (1 = disagree, 5 = agree)	Exercise.<br><br>Visual analogue scale, left side 'rarely exercised', right side 'several hours a day'	Mobility – Does the animal want to go for a walk?	Exercise. Describe your dog's exercise (where, when, how long, quality and freedom on walks)	During the last seven days, how often would you say that _____ has been taken for a walk?
**Interaction with humans or surroundings**	Please tell us how well this word describes your cat as he is today: Exploring.<br><br>7-point Likert scale (0 = Couldn't be more exploring, 6 = not at all exploring)	In the past 4 weeks my cat has been affectionate towards me	Thinking about just the last seven days…. my cat was curious about his/her surroundings/greeted me when I returned from being away<br><br>6-point Likert scale (1 = always, 6 = never)	Behaviour. Q3a. How interactive has your cat been during the past week?<br><br>6-point Likert scale (1 = not interactive at all, 6 = extremely interactive)	Happiness. My pet wants to play<br><br>5-point Likert scale (1 = disagree, 5 = agree)	Company of humans<br><br>Visual analogue scale, left side 'often alone', right side 'never without human company'	Happiness – is the pet responsive to things around it (e.g. family, toys)?	Companionship. Do you think a change to the amount and type of companionship your dog has would improve his/her wellbeing?<br><br>4-point Likert scale (1 = not at all, 4 = completely)	How often during the last seven days did you see _____ playing with toys?
**Appetite**		In the past 4 weeks my cat has had a healthy appetite	Thinking about just the last seven days…. my cat had a good appetite/enjoyed his food<br><br>6-point Likert scale (1 = always, 6 = never)	Eating. Q5a. How would you describe your cat's appetite during the past week?<br><br>4-point Likert scale (1 = normal, 4 = Not normal at all: he/she had a ravenous appetite or did not want to eat at all)		Food<br><br>Visual analogue scale, left side 'too fat/too thin', right side 'good weight and never hungry'	Hunger – is the pet eating enough?	Diet. Describe your dog's diet (what, when and where your dog eats and drinks)	Thinking about the last seven days, would you say that _____enjoyed his food?

Table S2. Examples of commonly included items for the four QoL assessment tools designed for cats.

Potentially valuable aspect of QoL	Noble et al (2019) [30]	Tatlock et al (2017) [31]	Freeman et al (2016) [16]	Bijsmans et al (2016) [26]
**Mood**	Please tell us how well this word describes your cat as he is today: Happy.<br><br>7-point Likert scale (0 = Couldn't be more happy, 6 = not all happy)	In the past 4 weeks my cat has appeared happy	Thinking about just the last 7 days….. my cat was irritable/friendly/relaxed/approachable	Behaviour. Q1a. How would you describe your cat's behaviour during the past week?<br><br>4-point Likert scale (1 = my cat seems happy; he/she behaves like a normal cat, 4 = my cat does not seem happy at all: he/she constantly behaves as if something is wrong)
**Grooming**		In the past 4 weeks my cat has been grooming itself as much as usual	Thinking about just the last 7 days….. my cat's coat or fur looked shiny/felt soft/looked dull/looked thick and full	Behaviour. Q6a. How has your cat's grooming been during the past week?<br><br>7-point Likert scale (1 = did not groom at all, 7 = grooming all the time)
